# Generalizability and treatment with sodium-glucose co-trasporter-2 inhibitors (SGLT2i) among patients with type 2 diabetes: an assessment using an Italian primary care database

**DOI:** 10.1007/s00592-024-02359-1

**Published:** 2024-08-29

**Authors:** Ippazio Cosimo Antonazzo, Davide Rozza, Paolo Angelo Cortesi, Carla Fornari, Elena Zanzottera Ferrari, Claire Paris, Caroline Eteve-Pitsaer, Marco Gnesi, Silvia Mele, Marco D’Amelio, Anna Rita Maurizi, Pasquale Palladino, Lorenzo Giovanni Mantovani, Giampiero Mazzaglia

**Affiliations:** 1 Research Centre on Public Health (CESP), University of Milano-Bicocca, Via Pergolesi 33, Monza, MB Italy; 2 Unit of Medical Statistics, Department of Clinical and Experimental Medicine, University of Pisa, Pisa, 56126 Italy; 3 Cegedim Health data, Milano, Italy; 4 Cegedim Health data, Boulogne-Billancourt, France; 5 Medical Evidence, Biopharmaceuticals Medical, AstraZeneca, Milan, Italy; 6 Value & Access, AstraZeneca, Milan, Italy; 7 Medical Affairs, Biopharmaceuticals Medical, AstraZeneca, Milan, Italy; 8 Laboratory of Public Health, IRCCS Istituto Auxologico Italiano, Milan, 20149 Italy

**Keywords:** Antidiabetic drug, Database research, Pharmaco-epidemiology, Primary care, SGLT2 inhibitors

## Abstract

**Aims:**

This study aimed to assess the proportions of type 2 diabetes (T2D) subjects meeting cardiovascular outcome trials (CVOTs) criteria for sodium-glucose cotransporter-2 inhibitors (SGLT-2i) and estimate SGLT2i utilization, along with associated demographic and clinical characteristics, in a primary care setting.

**Methods:**

T2D patients in Italy were selected between January 1, 2021, and December 31, 2022, from The Health Improvement Network (THIN^®^) database. Representativeness was determined by dividing patients meeting key inclusion criteria for four CVOTs (CANVAS, DECLARE-TIMI 58, EMPA-REG OUTCOME, VERTIS-CV) to the total T2D population. Demographic and clinical characteristics of eligible T2D subjects and SGLT2i users were compared, and logistic regression models assessed the likelihood of receiving SGLT2i.

**Results:**

Out of 17,102 T2D patients, 8,828 met eligibility criteria for at least one CVOT. DECLARE-TIMI 58 exhibited the highest representativeness (51.1%), compared to CANVAS (21.1%), EMPA-REG OUTCOME (5.5%), and VERTIS-CV (4.9%) trials. Eligible CVOTs patients were older (74.6 vs. 68.3 years), with a longer disease duration (10.2 vs. 9.7 years), and higher established cardiovascular disease (CVD) prevalence (36.0 vs. 27.3%) compared to SGLT2i users. Less than 10% of eligible T2D patients received SGLT2i. Males (OR: 1.43; 95%CI: 1.24–1.66) were more likely to be prescribed SGLT2i than other antidiabetic drugs, while the elderly (80 + vs. 40–64 years, OR: 0.17; 95% CI: 0.14–0.22) were less likely. Eligible T2D patients with CVD reported an increased likelihood of receiving SGLT2is compared to other antidiabetics.

**Conclusion:**

This study highlights significant variability in the proportion of T2D subjects meeting SGLT2i CVOT inclusion criteria, with DECLARE-TIMI-58 being the most represented. Low SGLT2i prescription rates in the Italian primary care setting, along with substantial demographic and clinical differences between SGLT-2i users and T2D eligible patients, emphasize the need for targeted interventions to optimize the use of these medications in primary care settings.

**Supplementary Information:**

The online version contains supplementary material available at 10.1007/s00592-024-02359-1.

## Introduction

Diabetes is a chronic metabolic condition that poses a significant global health threat. Findings from the Global Burden of Disease [1] indicate that in 2021 there were 529 million subjects worldwide living with diabetes, with a prevalence of 6.1%; these results in high health expenditures of US $966 billion globally, forecast to reach more than US $1054 billion by 2045. By 2050, more than 1.31 billion people are projected to have diabetes with an expected prevalence of around 10%.

Type 2 diabetes (T2D), generally associated with poor diet, obesity, and sedentary lifestyle, accounts for approximately 90% of all cases of diabetes and typically occurs in adults aged > 40 years. International guidelines advocate metformin and lifestyle changes as first measures for disease prevention, early management of T2D, as well as for preventing cardiovascular disease (CVD) occurrence. There is in fact evidence that improved glycemic control defers the onset of T2D, reduces its progression, and may partially reverse markers of microvascular complications [2, 3]. However, since T2D is a progressive disease, monotherapy is generally insufficient to maintain the glycemic targets in the long run and combination therapy is generally required.

The choice of medication added to the initial therapy is based on patients’ clinical characteristics and individual benefits. Important clinical characteristics include the presence of established CVD or cardiovascular (CV) risk factors, heart failure (HF), chronic kidney disease (CKD), as well as drug safety profile and costs.

In the last decade, sodium-glucose cotransporter 2 inhibitors (SGLT2i) emerged as a promising class of drugs that act by preventing the reabsorption of glucose from the proximal renal tubule in the kidney [4]. They have numerous pleiotropic effects such as reducing blood plasma glucose and blood pressure, weight, and inducing natriuresis, and they are currently considered the first-in-class treatment for T2D patients, particularly those with concomitant cardiovascular and/or renal comorbidities [5].

The efficacy of SGLT2i respect to glycemic control, and weight loss in patients with T2D is well documented. In recent years four cardiovascular outcome trials (CVOTs), namely EMPA-REG OUTCOME, CANVAS, VERTIS-CV and DECLARE-TIMI 58 [5–9], being conducted to assess the safety and efficacy of SGLT2i on cardiovascular endpoints in T2D patients with high CV risk, also demonstrated significant reduction in the occurrence of major CV endpoints and beneficial effects on renal outcomes.

However, randomized clinical trials (RCT) often include highly selected and homogenous population to achieve internal validity and maximize the discriminatory power of drug effects [10, 11]. The generalizability of clinical trials results to real clinical practice is recognized as a major issue because strict selection of participants might impair the applicability of trial results to a more general population with benefits and harms from a treatment found in RCTs being an over- or underestimation of the real-world outcomes. This is often evaluated as external validity of the study as it is important in clinical decision making and in implementing clinical guidelines. An understanding of external validity becomes of utmost relevance among SGLT2-i CVOTs because most of the recent guidelines has strengthened the role of SGLT2i in the management of T2D patients [12].

To quantify the generalizability of SGLT2i findings from CVOTs to real-world patients, studies from Europe [13–15], US [16], and Taiwan [17] have been conducted in which trial inclusion and exclusion criteria have been applied to populations of T2D patients. All studies found large differences between trials regarding the proportion of patients seen in clinical practice that would have met entry criteria in these CVOTs, with the DECLARE-TIMI 58 trial consistently reported as the most generalizable and applicable one [18].

However, few data still exist on the differences between patients eligible to the SGLT2-i CVOTs and those using SGLT2-i in a real-world setting. Hinton et al., [15] reported that only ~ 10% of those who meet the study criteria are currently prescribed SGLT2i although it is not clear what patients’ characteristics are associated with physicians’ decision of using these medications. In Italy the possibility among GPs to prescribe SGLT2is in T2D under NHS reimbursement has been opened since 2022, under the recommendations issue by the Italian Medicine Agency (AIFA) [19]. We, therefore, assessed the proportions of adults with T2D in the Italian primary care setting who would have met enrolment criteria for CVOTs of the four marketed SGLT2i, and we estimated prevalence and clinical characteristics associated to SGLT2i use among T2D patients eligible to the CVOTs.

## Materials and methods

### Study design and data source

We conducted a cross-sectional analysis of adults with T2D included the list of Italian general practitioners (GPs) participating to The Health Improvement Network (THIN^®^). THIN is a large standardized European network of databases of fully anonymized electronic medical records collected from general practices that agreed to participate in the network and it is compliant with the current EU general data protection regulation (GDPR). The database consists of coded information on patient characteristics, drug prescriptions, diagnoses, consultations, diagnostic test results, and referrals to secondary care [20, 21].

In Italy, THIN gathers longitudinal anonymous patient-level information on healthcare services reimbursed by the Italian National Healthcare System (NHS). It encompasses approximately 1 million active patients with an average of around 7 years of clinical data history, registered with over 500 Italian GPs located throughout the country. Symptoms and diagnoses are coded according to the International Classification of Diseases, Ninth Revision, Clinical Modification (ICD-9 CM), whereas drug prescriptions are classified using the Anatomical Therapeutic Chemical (ATC) classification.

### Study population

All patients actively registered (i.e., at least one contact with the GP for any medical or administrative reason in the study period) in the list of participating GPs between January 1, 2021, and December 31, 2022, with an available follow-back of at least five years prior to the index date (ID) and at least one measurement of HbA1c were considered. In our study, the date of the first contact in the study period was defined as the ID.

Patients with T2D aged > 18 year were identified if they met at least one of the following criteria: (1) diagnosis of diabetes (ICD-9CM: 250 excluding evidence of type 1 diabetes or gestational diabetes), (2) clinical investigations (Fasting serum glucose ≥ 126 mg/dl and HbA1c > 6.5%), and (3) glucose-lowering medications (ATC: A10).

To identify groups of people who would meet the criteria for inclusion in each of the four SGLT2i CVOTs, we used the nearest matching ICD-9CM codes available to determine cardiovascular risk factors. These include codes for diagnosis, procedures, and laboratory-based data. Wider criteria were adapted for those conditions that are not recorded in primary care (Supplementary data). For example, for hospital admission for unstable angina, we used diagnosis codes for unstable angina and codes indicating poor angina control.

### Patients’ characteristics

The demographic and clinical characteristics of T2D patients were evaluated with regard to age, gender, diabetes duration and microvascular complication, presence of cardiovascular diseases (i.e., heart failure, coronary heart disease, atrial fibrillation, stroke/TIA, peripheral artery disease), chronic kidney disease, presence of hypertension, Charlson comorbidity index [22, 23], BMI, HbA1c, blood pressure (BP), cholesterol levels, and number of different concomitant therapies. Age was defined at the ID, BMI was taken as the last value recorded prior to ID, whereas HbA1c, eGFR, BP, and cholesterol levels was taken as the last recorded measurement within the six months before the ID. Comorbid conditions and diabetes complications were captured as recorded diagnoses any time prior to the ID. Duration of diabetes was determined from the first event that indicated diabetes, which included diagnosis codes, investigation results to indicate diabetes, or the date for a first prescription of a glucose-lowering drug and the ID. Finally, to capture data on currently used medications, we took the most recent prescriptions in the 6 months before the ID.

### Study outcomes

For each CVOT trial, eligibility was determined by dividing the number of patients fulfilling the respective key inclusion and exclusion criteria by the total enrolled T2D population. The proportion of eligible patients using SGLT2is was calculated by considering the prescription of at least one SGLT2i within six months after the ID.

### Statistical analysis

Eligibility was calculated as a prevalence, as described above. For each different trial we described demographic and clinical characteristics of eligible patients, using proportions for categorical variables, and mean (SD) or median (IQR) for continuous variables. Subsequently, we reported any demographic and clinical difference between the population eligible to start this class of medicines and those being prescribed at least one SGLT2i. Chi-square test for proportions and t-tests for continuous variables were used to detect any possible difference (*P* < 0.05). We evaluated the proportion of individuals treated with SGLT2is among individuals eligible to the respective CVOT. Additionally, we evaluated the proportion of individuals who met the inclusion criteria for the different CVOT trial among individuals treated with the corresponding SGLT2i treatment. Finally, among individuals who were potentially eligible to at least one CVOT trial, a multivariate logistic regression model was employed to assess the impact of demographic and clinical characteristics on the likelihood of receiving a SGLT2i compared with other antidiabetic drugs. Results were expressed as Odds Ratio (OR) with 95% confidence intervals (95%CI).

## Results

**Fig. 1:**
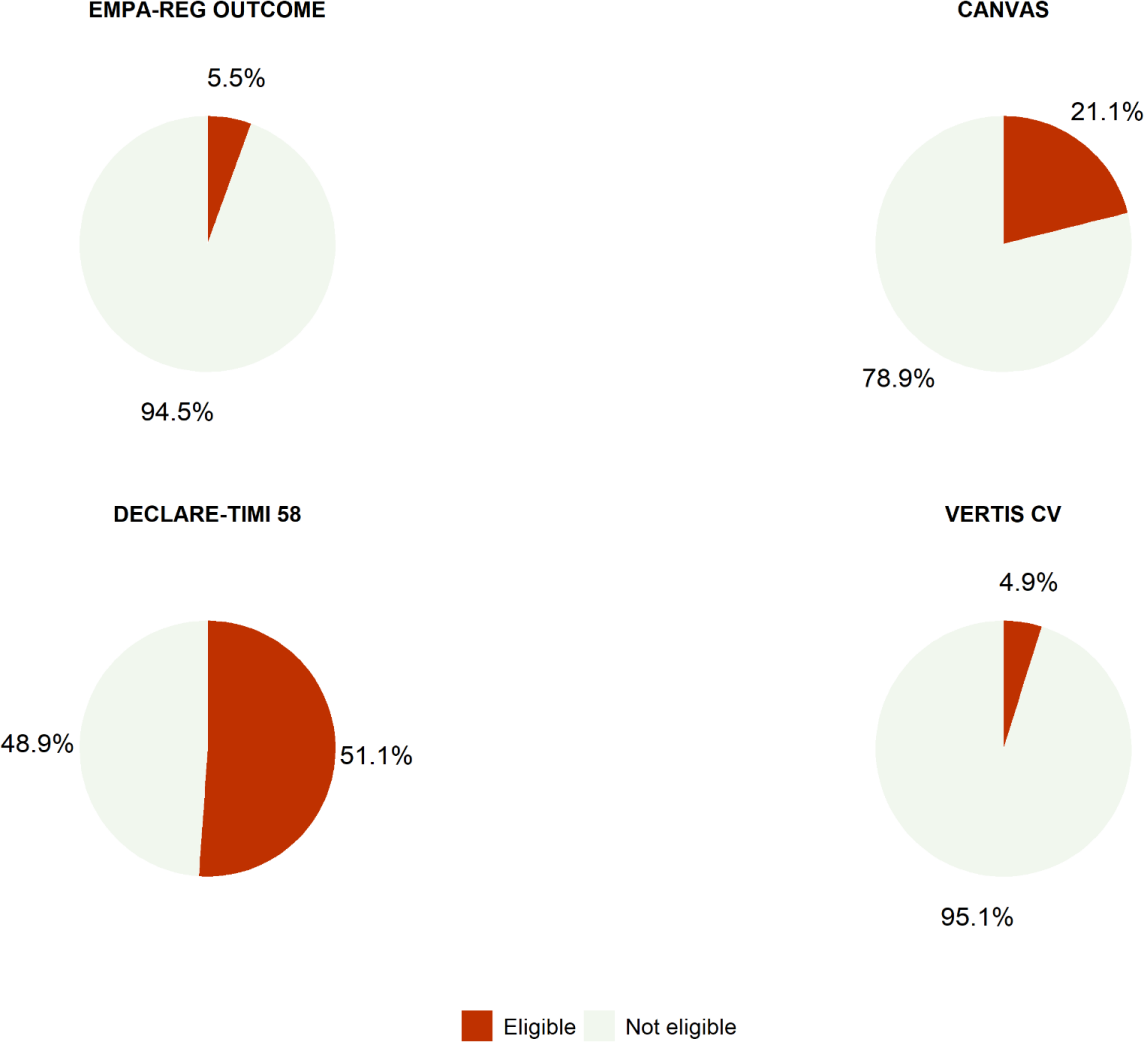
Percentages of adults with T2D in the THIN database who would have met inclusion criteria for the CVOTs with empagliflozin (EMPA-REG Outcome), canagliflozin (CANVAS), dapagliflozin (DECLARE-TIMI 58), or ertugliflozin (VERTIS CV)

During the study period, 17,102 patients with T2D were selected. Overall, 8,828 patients met the eligibility criteria for at least one CVOT. Among them, 8,741 (51.1%) would have met the criteria of eligibility for dapagliflozin, 3,614 (21.1%) for canagliflozin, 949 (5.5%) for empagliflozin, and 830 (4.9%) for ertugliflozin (Fig. 1).

Participants in the CVOTs were younger than people in THIN database (EMPA-REG 63.1 vs. 75.5 years; CANVAS 63.3 vs. 75.1 years; DECLARE 63.8 vs. 74.8 years; VERSIT-CV 64.4 vs. 75.6 years), with lower BMI (EMPA-REG 28.5 vs. 30.6; CANVAS 29.1 vs. 31.9; DECLARE 29.2 vs. 32.1; VERSIT-CV 28.5 vs. 32.0), and lower eGFR levels (EMPA-REG 71.4 vs. 74; CANVAS 72.7 vs. 76.5; DECLARE 75 vs. 86.1; VERSIT-CV 71.2 vs. 76) (Table 1). Conversely, we observed longer duration of diabetes (with the exception of CANVAS) and higher levels of HbA1c. Due to the specific eligibility criteria, EMPA-REG and VERTIS-CV shared similar proportion of subjects with established CVD compared to THIN, whereas for DECLARE and CANVAS a higher proportion of participants with established CVD was observed in the CVOTs’ participants.

**Table 1:** Demographic and clinical characteristics of T2D patients enrolled in the CVOTs and those with T2D in the THIN database eligible for the trials

	EMPA-REG (Empagliflozin)		CANVAS (Canagliflozin)		DECLARE-TIMI 58 (Dapagliflozin)		VERTIS-CV (Ertugliflozin)	
	THIN eligible [*N* = 949]	EMPA-REG participants [*N* = 7,034]	THIN eligible [*N* = 3,614]	CANVAS participants [*N* = 10,142]	THIN eligible [*N* = 8,741]	DECLARE-TIMI 58 [*N* = 17,160]	THIN eligible [*N* = 830]	VERTIS [*N* = 8,238]
**Gender (Males)**	63.9%	71.5%	55.8%	64.9%	54.8%	62.6%	64.6%	70%
**Mean Age**	75.5 (9.1)	63.1 (8.6)	75.1 (9.1)	63.3 (8.3)	74.8 (8.9)	63.8 (6.8)	75.6 (9.2)	64.4 (8.1)
**Diabetes duration (years)**,** mean (SD)**	11.5 (7.7)	17.3 (11.6)	14.3 (6.3)	13.5 (7.7)	10.2 (6.7)	11.8 (7.8)	11.5 (7.7)	12.9 (8.3)
**Cardiovascular disease §**	**99.8%**	**99.9%**	**42.1%**	**65.6%**	**36.4%**	**40.6%**	**99.8%**	**99.9%**
*Coronary heart disease****°***	40.4%	75.6%	13.6%	55.8%	9.5%	33.0%	37.0%	76.3%
*Stroke*	59.6%	23%	15.7%	12.8%	9.3%	6.5%	62.5%	21%
*PAD*	10.9%	21%	2.9%	20.8%	1.5%	6.0%	12.7%	18.8%
**Heart failure**	12.1%	10.1%	8.4%	14.4%	8.6%	9.9%	11.6%	23.1%
**Chronic kidney disease a**	29.9%	25.5%	27.2%	19.8%	25.3%	9.1%	30.0%	21.6%
Vital signs [mean (SD)] *#								
*BMI*	28.5 (4.4)	30.6 (5.3)	29.1 (5.4)	31.9 (5.9)	29.2 (5.4)	32.1 (6.0)	28.5 (4.9)	32.0 (5.4)
*Blood pressure Diastolic*	77.1 (10.2)	77 (10)	77.6 (9.4)	77.6 (9.6)	78.2 (9.8)	78.0 (9.1)	76.8 (10.1)	77 (8.5)
*Blood pressure Systolic*	134.7 (16.2)	135 (17)	136.1 (16.2)	136.4 (15.8)	136.1 (16.5)	135.1 (15.3)	134.6 (16.0)	133 (13.8)
*Tobacco smoking ^*	23.4%	13.0%	17.0%	17.8%	22.7%	14.5%	24.7%	NR
Laboratory test [mean (SD)] *#								
*HbA1c (%)*	7.5 (0.5)	8.1 (0.8)	7.6 (0.5)	8.2 (0.9)	7.2 (0.6)	8.3 (1.2)	7.5 (0.5)	8.3 (0.9)
*TOTAL Cholesterol (mg/dL)*	161.2 (26.8)	162.2 (42.5)	164.6 (26.9)	170.1 (46.4)	168.1 (27.4)	169.8 (46.3)	161.1 (26.4)	169 (46.5)
*LDL Cholesterol (mg/dL)*	85.2 (23.7)	84.9 (34.8)	87.9 (22.9)	88.9 (34.8)	90.5 (23.3)	88.8 (34.7)	85.2 (23.6)	89 (38.3)
*HDL Cholesterol (mg/dL)*	48.3 (8.9)	46.3 (11.6)	49.2 (8.9)	46.4 (11.6)	49.4 (8.9)	46.3 (11.6)	48.3 (8.8)	44 (12.1)
*eGFR*	71.7 (26.4)	74 (21)	72.7 (29.4)	76.5 (20.5)	75.0 (29.0)	86.1 (21.8)	71.2 (27.5)	76.0 (20.9)

**§** Patients with at least one of the following: CHD, angina, MI, stroke, and PAD

° includes MI, CABC/PCI, Angina

a eGFR<60

* Unless otherwise stated

**^** % on total patients with registered information

**#** Patients with registered information (% on total diabetics): BMI (51.5%); DBP (72.3%); SBP (51.6%); smoking status (72.5%); HbA1c (100%); total cholesterol (68.7%); LDL cholesterol (61.1%); HDL cholesterol (66.3%); eGFR (39.2%)

Table 2 explores the differences in demographic and clinical characteristics of T2D patients eligible to at least one CVOT and those prescribed with at least one SGLT2i being retrieved in the THIN database. Compared to T2D patients eligible to CVOTs, SGLT2i users were younger (68.3 vs. 74.6 years), with shorter disease duration (9.7 vs. 10.2 years) and with lower prevalence of established CVD (27.3% vs. 36.0%). Lower comorbidity was reported not only for CVD but also for other diseases as demonstrated by the Charlson index, where the proportion of SGLT2i users with a score > 2 was significantly lower than among those eligible to CVOTs (21.1% vs. 29.2%).

**Table 2:** Demographic and clinical characteristics of patients with type 2 diabetes in the THIN database who would have met inclusion criteria for at least one of the CVOTs trials and of those who has been prescribed at least one SGLT2i

	Eligible to CVOTs [*N*=8832]	SGLT2i users [*N*=1594]	*P*-value
**Sex**			<0.001
*Male*	4826 (54.6%)	1022 (64.1%)	
*Female*	4006 (45.4%)	572 (35.9%)	
**Age**,** mean (SD)**	**74.6 (9.1)**	**68.3 (9.4)**	**<0.001**
***Disease duration (years)***,*** mean (SD)***	10.2 (6.7)	9.7 (6.5)	<0.001
*0–7*	3798 (43.0%)	757 (47.5%)	
*8+*	5033 (57.0%)	837 (52.5%)	
**Cardiovascular disease §**	**3183 (36.0%)**	**435 (27.3%)**	**<0.001**
*Coronary heart disease*	830 (9.4%)	161 (10.1%)	<0.001
*Stroke*	814 (9.2%)	90 (5.6%)	<0.001
*PAD*	133 (1.5%)	18 (1.1%)	0.006
**Heart failure**	750 (8.5%)	113 (7.1%)	<0.001
**Atrial fibrillation**	1102 (12.5%)	148 (9.3%)	<0.001
**Hypertension**	**7950 (90.0%)**	**1056 (66.2%)**	**<0.001**
**Chronic Kidney Disease**	**2222 (25.2%)**	**359 (22.5%)**	**<0.001**
**Charlson index**			<0.001
*Mild (1–2)*	6261 (70.9%)	1261 (79.1%)	
*Moderate (3–4)*	2328 (26.4%)	309 (19.4%)	
*Severe (5+)*	243 (2.8%)	24 (1.5%)	
Vital signs [mean (SD)] *#			
*BMI*	29.3 (5.5)	29.6 (5.8)	0.656
*Obese (30+) ^*	1989 (38.2%)	377 (41.1%)	0.45
*Blood pressure Diastolic*	78.3 (9.8)	79.2 (11.1)	0.003
*Blood pressure Systolic*	136.1 (16.5)	135.0 (17.4)	0.076
*Tobacco smoking ^*	688 (22.6%)	103 (22.8%)	0.233
Laboratory test [mean (SD)] *#			
*HbA1c (%)*	7.2 (0.6)	7.3 (0.7)	0.024
*Uncontrolled (7+) ^*	5126 (58.0%)	1036 (65.0%)	0.106
*TOTAL Cholesterol (mg/dL)*	168.2 (27.4)	166.7 (26.9)	0.237
*LDL Cholesterol (mg/dL)*	90.6 (23.3)	88.9 (22.6)	0.501
*HDL Cholesterol (mg/dL)*	49.4 (8.9)	48.7 (8.8)	0.435
*eGFR*	75.4 (29.4)	85.0 (31.1)	<0.001
**N. Concomitant therapies**			<0.001
*0–4*	3949 (44.7%)	728 (45.7%)	
*5–9*	2556 (28.9%)	450 (28.2%)	
*≥ 10*	2327 (26.3%)	416 (26.1%)	
**Therapeutic class**			
*Antiplatelet agents*	3581 (40.5%)	607 (38.1%)	<0.001
*ACEis/ARNI*	2620 (29.7%)	419 (26.3%)	<0.001
*ARBs*	2759 (31.2%)	463 (29.0%)	<0.001
*Beta blockers*	3275 (37.1%)	564 (35.4%)	<0.001
*Diuretics*	1840 (20.8%)	265 (16.6%)	<0.001
*Lipid-lowering agents*	4271 (48.4%)	830 (52.1%)	0,427

**§** patients with at least one of the complications/diseases mentioned below

* unless otherwise stated

**^** N & % on total patients with registered information

**#** patients with registered information (% on total diabetics): BMI (51.5%); DBP (72.3%); SBP (72.5%); smoking status (27.5%); HbA1c (100%); total cholesterol (68.7%); LDL cholesterol (61.1%); HDL cholesterol (66.3%); eGFR (39.2%); albuminuria (34.4%); UACR (4.8%)

**Fig. 2:**
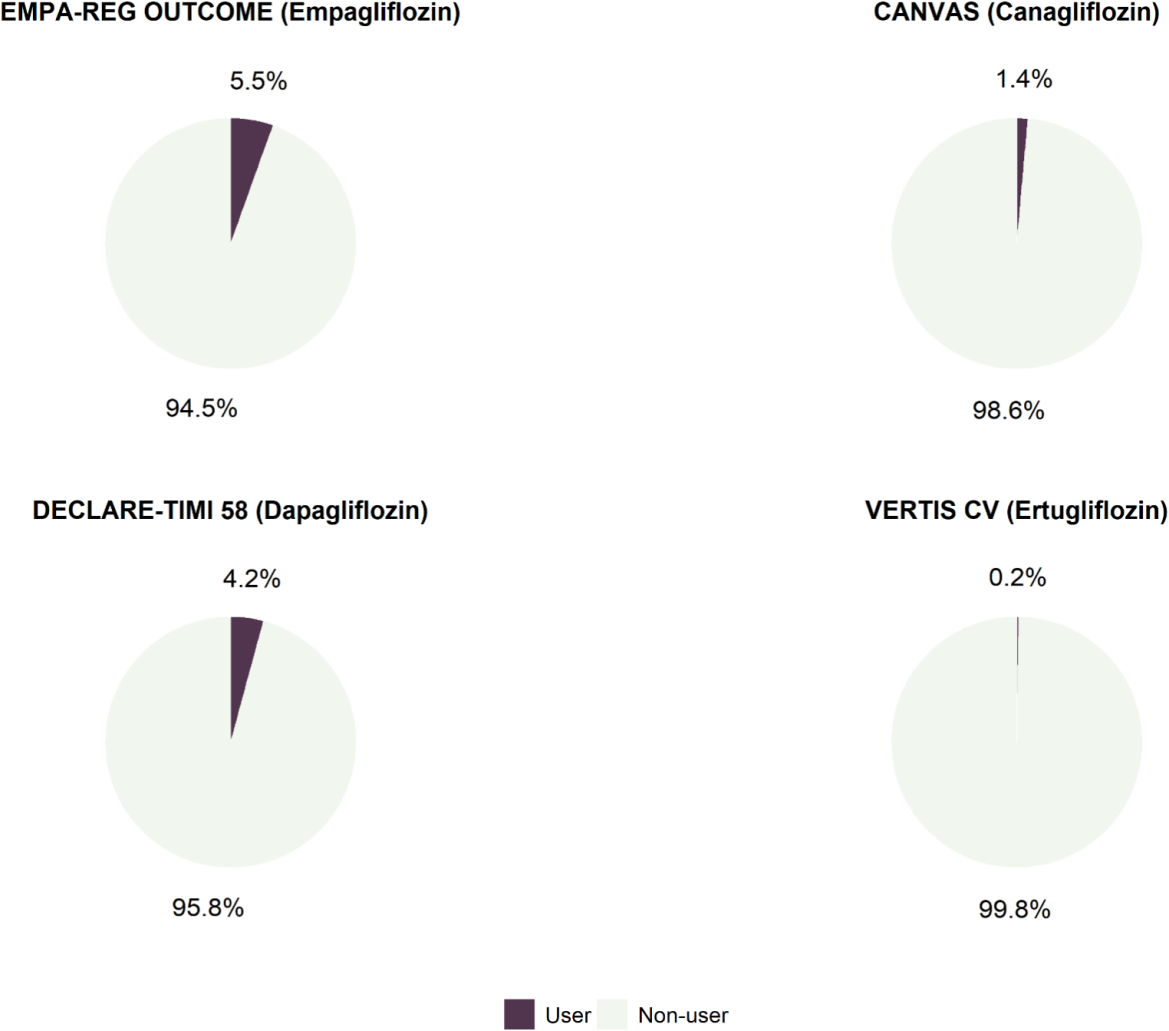
Percentages of subjects treated with empagliflozin, canagliflozin, dapagliflozin, or ertugliflozin among those eligible to the respective CVOT

Figure 2 reports the percentage of patients treated with the different SGLT2is among those who were eligible for the different CVOTs trial. Overall, less than 10% of eligible patients were treated with SGLT2is. Specifically, the highest prevalence was observed among patients potentially eligible for the EMPA-REG OUTCOME trial, of whom 5.5% were also treated with empagliflozin, whilst the lowest prevalence was observed among patients eligible for the VERTIS-CV trial (0.2% treated with ertugliflozin). Among the study cohort, the percentage of patients eligible to different CVOTs among those treated with different SGLT2is ranged from 2.2 to 40.6%. Specifically, the lowest value was observed for patients eligible to VERTIS CV among those treated with ertugliflozin (2.2%), and the highest value was observed for those eligible to DECLARE-TIMI 58 among those treated with dapagliflozin (40.6%) (Fig. 1 supplementary material).

**Fig. 3:**
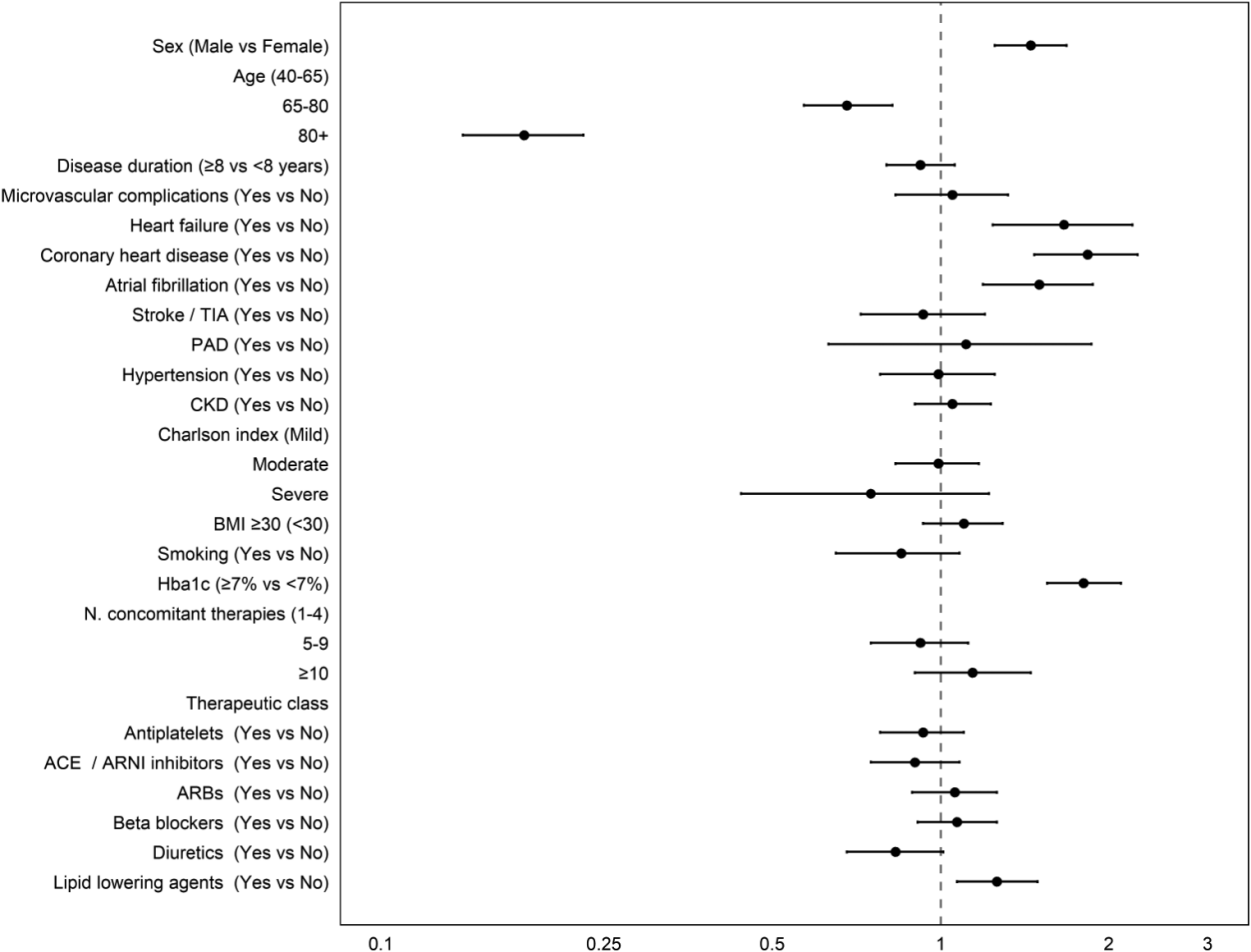
Multivariable logistic regression to assess demographic and clinical factors associated with prescribing SGLIT2i vs. other antidiabetic therapies in patients potentially eligible to CVOTs trials. *CKD: patients with a diagnosis code of Chronic kidney disease or patients with eGFR ≤90 ml/min*

Results from univariable and multivariable models exploring factors associated with SGLT2is drug use compared other antidiabetics are reported in Fig. 3 and Supplementary Table 2. Results indicated that males were more likely to be prescribed SGLT2is therapies compared with females (OR: 1.43; 95%CI: 1.24–1.66). On the contrary, the likelihood of being prescribed with SGLT2is was lower among elderly people (80 + vs. 40–64 years, OR: 0.17; 95% CI: 0.14–0.22). T2D patients having the following comorbidities reported an increased likelihood of receiving SGLT2is compared to other antidiabetics: heart failure (OR: 1.56; IC95%: 1.17–2.05), coronary heart disease (OR: 1.82; IC95%: 1.47–2.24), and atrial fibrillation (OR: 1.49; IC95%: 1.20–1.84). Finally, patients with uncontrolled HbA1c were significantly more likely to be prescribed SGLT2is compared with those with lower HbA1c values (OR: 1.79; IC95%: 1.54–2.08).

## Discussion

This is the first population-based study estimating the proportion of patients with T2D that would have been eligible for inclusion in the SGLT2i-CVOTs in the Italian primary care setting. Findings from the present study confirm what emerged from previous studies [13–18], which reported the highest representativeness for DECLARE-TIMI 58 trial (50%), almost two-fold higher than CANVAS (21.1%) and even more higher than EMPA-REG OUTCOME (5.5%) and VERTIS-CV (4.9%). These results are not surprising considering how the four CVOTs used different inclusion criteria related to baseline CV. In fact, two of the trials included only patients with established CV disease (EMPA-REG OUTCOME and VERTIS-CV), while the other two trials also included patients with additional CV risk factors (DECLARE-TIMI 58, ≥ 2 CV risk factors, and CANVAS, ≥ 3 CV risk factors). This suggests that the main inclusion criteria, determining the baseline CV risk in the respective CVOT, are the most relevant drivers for determining how representative the trials were of a general T2D population.

Our results are also consistent with a study conducted among Italian diabetologists [18], where DECLARE-TIMI 58 and CANVAS reported higher proportion of eligible T2D patients. In this study, the proportion of eligible subjects was slightly higher for all CVOTs; these findings might be explained by differences in data sources and T2D patients’ severity, since diabetologists often manage patients with more advanced and complex T2D cases than GPs.

Results from this study also demonstrate that, in line with results from previous studies [15], less than 10% of eligible patients were treated with SGLT2is. In addition, we have observed significant differences between T2D subjects eligible to at least one CVOT and SGLT2i users, with the latest being younger, with less disease duration and lower comorbidities. Several factors may contribute to these findings. First, efficacy of SGLT2i in glycemic control is influenced by renal function; all SGLT2i are indicated in adults for the treatment of CKD with and without T2DM because they prevent decline in kidney function through reduction in glomerular hypertension mediated through tubule-glomerular feedback, but also among T2D patients with impaired kidney function independent of their effect on glycemic control [24, 25]. However, the guidelines issued by AIFA [19] suggest using all SGLT2i (with or without dose adjustment) in subjects with eGFR > 30 mL/min, whereas only dapagliflozin and canagliflozin were recommended in patients with eGFR < 30 mL/min. Therefore, it is possible to speculate that clinicians may be less prone in using SGLT2i in patients with T2D and impaired kidney function. This may explain our findings where SGLT2i users reported significantly higher levels of eGFR compared to those eligible to CVOTs (85.0 vs. 75.4 mL/min vs.; *p* < 0.001), and patients with diagnosed chronic kidney disease were not more likely to be prescribed with SGLT2i compared to other antidiabetic drugs.

Second, recently issued guidelines from the Italian Medicines Agency (AIFA) on the use of SGLT2i [19] for reimbursement purposes recommended the use of these medications as second-line treatment as an add on or to replace metformin with a specific focus to improve blood glucose levels. In addition, the same guidelines have acknowledged the positive findings of CVOTs and recommend these medications for people with established CVD or with high CV risk. Again, this is reflected in our findings which demonstrated preferential use of SGLT2i compared to other antidiabetics in T2D patients affected by heart failure (OR: 1.56; 95% CI: 1.18–2.06), CHD (OR: 1.82; 95% CI: 1.18–2.06), atrial fibrillation (OR: 1.49; 95% CI: 1.20–1.85), as well as in patients with uncontrolled blood glucose. Conversely, elderly patients were less likely to be prescribed with SGLT2i. This behavior among Italian GPs might be explained by the fact that elders are patients more likely to be affected by T2D for a longer time. Therefore, in presence of stable glycemic control among these patients, GPs may tend to maintain antidiabetic medications commonly used before the introduction of SGLT2i. In this context, future studies should be conducted to assess whether the new AIFA regulation on the use of SGLT2i impacts on prescription behavior of the Italian GPs. In addition, in light of the efficacy showed by SGLT2i and glucagon-like-peptide-1 receptor agonists (GLP1ra) in improving cardiovascular outcomes in patients with T2D and in those with high risk for cardiovascular disease [26], future studies should be conducted to investigate the impact of national and international guidelines on prescription patterns of these therapeutic drug classes in GPs setting.

The data source used to identify T2D patients according to the inclusion criteria of each CVOT carries several limitations. First, there is limited information on laboratory measurements, particularly urine albumin, hepatic enzymes, which is the main reason why we decided to define eligibility only on the basis of inclusion criteria; this, in turns, could have led to an overestimation of CVOT eligibility for the general population of patients with T2D. Second, because of the nature of the data source, it is likely that the number of people identified was underestimated for some variables. This may explain the lower prevalence of CHD observed in eligible patients compared to the CVOTs, considering that procedures like CABG or PCI are not requested by GPs, and there may be a lag in case ascertainment in primary care. Third, THIN contains medication records based on prescriptions, but it is not known whether the prescribed medications were taken by patients. Nonetheless, a validation study has confirmed that THIN data are effective in producing reliable results in drug patterns, particularly for chronic treatments [27].

## Conclusion

This study, utilizing the primary care data, reveals substantial variability in the external validity of the different CVOTs of SGLT2i within the T2D population. Remarkably, DECLARE-TIMI-58 emerges as the most representative, encompassing nearly 50% of eligible patients in contrast to other CVOTs. These differences primarily stem from variations in the baseline cardiovascular risk criteria employed in these trials. The study highlights that only a minority of eligible patients actually received SGLT2is. These findings can be attributed to multiple factors, including guidelines from the Italian medicine agency. Additionally, the beliefs and awareness of general practitioners, who historically deferred such prescription to diabetologists, may contribute to this observed phenomenon. Our findings emphasize the need for enhancing GP’s awareness of SGLT2is and prompt further investigations to evaluate potential shifts in drug prescription practices within primary care.

## Electronic supplementary material

Below is the link to the electronic supplementary material.

Supplementary Material 1

## Data Availability

The data used in the preparation of this article are available from the Cegedim company upon reasonable request.
