# Supplementary material for: Generalizability and treatment with sodium-glucose co-trasporter-2 inhibitors (SGLT2i) among patients with type 2 diabetes: an assessment using an Italian primary care database
Source: Acta Diabetol. 2024 Aug 29;62(3):343–51. doi: 10.1007/s00592-024-02359-1 (PMC11872765; doi:10.1007/s00592-024-02359-1)

**Title:** Generalizability and treatment with sodium-glucose co-trasporter-2 inhibitors (SGLT2i) among patients with Type 2 diabetes: An assessment using an Italian primary care database

**Authors:** Ippazio Cosimo Antonazzo 1, 2, Davide Rozza 1, Paolo Angelo Cortesi 1, Carla Fornari 1, Elena Zanzottera Ferrari 3, Claire Paris 4, Caroline Eteve-Pitsaer 4, Marco Gnesi 5, Silvia Mele 6, Marco D’Amelio 6, Anna Rita Maurizi 7, Pasquale Palladino 3, Lorenzo Giovanni Mantovani 1, Giampiero Mazzaglia 1

**Affiliation:**

1. Research Centre on Public Health (CESP), University of Milano-Bicocca, Monza, Italy.
2. Unit of Medical Statistics, Department of Clinical and Experimental Medicine, University of Pisa, 56126 Pisa, Italy.
3. Cegedim Health data, Milano, Italy.
4. Cegedim Health data, Boulogne-Billancourt, France.
5. Medical Evidence, Biopharmaceuticals Medical, AstraZeneca, Milan, Italy
6. Value & Access, AstraZeneca, Milan, Italy
7. Medical Affairs, Biopharmaceuticals Medical, AstraZeneca, Milan, Italy

**Corresponding**

Carla Fornari

Centre on Public Health (CESP), University of Milan-Bicocca,

Via Pergolesi 33, Monza (MB), Italy.

carla.fornari@unimib.it

**Supplementary material**

**Supplementary Table 1.** Description of inclusion criteria for respective CVOT and the method of selection in THIN

| **Inclusion criteria** | **DECLARE-TIMI 58 (dapagliflozin)** | **EMPA-REG OUTCOME (empagliflozin)** | **CANVAS (canagliflozin)** | **VERTIS (ertugliflozin)** |
| --- | --- | --- | --- | --- |
| **Age** | ≥ 40 and history of CVD, or ≥ 55 (males) or ≥ 60 (females) and with CV risk factors | ≥18 | ≥ 30 and history of CVD, or ≥ 50 and with CV risk factors | ≥40 |
| **HbA_1c_ (%)** | ≥6.5% to <12% | ≥7.0% to <9.0% | ≥7.0% to <10.5% | ≥7.0% to <10.5% |
| **Renal function** (eGFR (ml min_1 1.73 m_2)>30 | No criterion | Nor diagnosis of acute or chronic kidney failure neither registered value of eGFR <30 12 months prior to ID | Nor diagnosis of acute or chronic kidney failure neither registered value of eGFR <30 12 months prior to ID | No criterion |
| **History of CVD (≥ 1 of the following)** |  |  |  |  |
| o   CHD |  |  |  |  |
| *§ Documented MI* | Coded diagnosis*: 410 | Coded diagnosis*: 410 | Coded diagnosis*: 410 | Coded diagnosis*: 410 |
| *§ PCI/ coronary revascularization* | Coded procedures*: 00.66, 36.03, 36.09, V45.82 | Coded procedures*: 00.66, 36.03, 36.09, V45.82 | Coded procedures*: 00.66, 36.03, 36.09, V45.82 | Coded procedures*: 00.66, 36.03, 36.09, V45.82 |
| *§ Coronary artery bypass (CABG)* | Coded diagnosis & procedures*: 414.02-07, V45.81-82 | No criterion | Coded diagnosis & procedures*: 414.02-07, V45.81-82 | Coded diagnosis & procedures*: 414.02-07, V45.81-82 |
| *§ Angina* | Coded diagnosis*: 411, 413, 414.01 | Coded diagnosis*: 411, 413, 414.01 | Coded diagnosis*: 411, 413, 414.01 | No criterion |
| o   Cerebrovascular disease |  |  |  |  |
| *§ Stroke* | Coded diagnosis*: 433-434 (excl. 43X.X0), 436, V12.54 | Coded diagnosis*: 430-432, 433-434 (excl. 43X.X0), 436, V12.54 | Coded diagnosis*: 430-432, 433-434 (excl. 43X.X0), 436, V12.54 | Coded diagnosis*: 433-434 (excl. 43X.X0), 436 |
| o   Peripheral artery disease |  |  |  |  |
| *§  Peripheral arterial intervention, stenting, or surgical revascularization* | Coded diagnosis*: 440.2, 440.20, 440.21,  440.22, 440.23, 440.24, 440.29, 440.3, 440.30,  440.31, 440.32, 443.81, 433.9, 444.2 | Coded diagnosis*: 440.2, 440.20, 440.21,  440.22, 440.23, 440.24, 440.29, 440.3, 440.30,  440.31, 440.32, 443.81, 433.9, 444.2 | Coded diagnosis*: 440.2, 440.20, 440.21,  440.22, 440.23, 440.24, 440.29, 440.3, 440.30,  440.31, 440.32, 443.81, 433.9, 444.2 | Coded diagnosis*: 440.2, 440.20, 440.21,  440.22, 440.23, 440.24, 440.29, 440.3, 440.30,  440.31, 440.32, 443.81, 433.9, 444.2 |
| *§ Lower extremity amputation* | Coded procedures*: 84.11-84.19 | Coded procedures*: 84.11-84.19 | Coded procedures*: 84.11-84.19 | Coded procedures*: 84.11-84.19 |
| **CV Risk factors** |  |  |  |  |
| o   Dyslipidaemia | Coded diagnosis*: 272 | No criterion | Coded diagnosis*: 272 | No criterion |
| o   BMI | No criterion | BMI≤45 | No criterion | BMI≥18 |
| o   Hypertension | Coded diagnosis*: 401-405, 997.91  AND ≥ 1 anti-hypertensive  agents 6 months prior to index date | No criterion | Coded diagnosis*: 401-405, 997.91  AND ≥ 1 anti-hypertensive  agents 6 months prior to index date | No criterion |
| o   Tobacco use | Evidence of current smoking (yes/no) 12 months prior to index date | No criterion | Evidence of current smoking (yes/no) 12 months prior to index date | No criterion |
| o   Disease duration > 10 years | No criterion | No criterion | ≥10 | No criterion |

* ICD9-CM

**Supplementary Table 2.** Crude and multivariable logistic regressions to assess demographic and clinical factors associated with prescribing SGLT2i Vs Other antidiabetic therapies in patients potentially eligible to CVOTs trials.

|  | **Crude**  **OR (95%CI)** | **P-value** | **Adjusted**  **OR (95%CI)** | **P-value** |
| --- | --- | --- | --- | --- |
| *Sex (Male)* | *1.65 (1.44, 1.90)* | *<0.001* | *1.45 (1.25, 1.68)* | *<0.001* |
| *Age* |  |  |  |  |
| *40-65* | *Reference* | *Reference* | *Reference* | *Reference* |
| *65-80* | *0.68 (0.58, 0.80)* | *<0.001* | *0.68 (0.57, 0.82)* | *<0.001* |
| *80+* | *0.19 (0.15, 0.24)* | *<0.001* | *0.18 (0.14, 0.23)* | *<0.001* |
| *Diabetes duration (≥ 8 years)* | *0.89 (0.78, 1.02)* | *0.095* | *0.92 (0.80, 1.06)* | *0.240* |
| *Microvascular complications (Yes)* | *1.15 (0.93, 1.42)* | *0.199* | *1.05 (0.83, 1.32)* | *0.662* |
| *Heart failure* | *1.40 (1.08, 1.80)* | *0.008* | *1.66 (1.24, 2.20)* | *<0.001* |
| *Coronary heart disease* | *1.84 (1.51, 2.23)* | *<0.001* | *1.83 (1.47, 2.25)* | *<0.001* |
| *Atrial fibrillation* | *1.16 (0.95, 1.41)* | *0.137* | *1.50 (1.19, 1.87)* | *<0.001* |
| *Stroke / TIA* | *0.89 (0.72, 1.11)* | *0.309* | *0.93 (0.72, 1.20)* | *0.607* |
| *PAD* | *1.18 (0.69, 1.92)* | *0.522* | *1.11 (0.63, 1.86)* | *0.712* |
| *Hypertension* | *0.82 (0.66, 1.01)* | *0.061* | *0.99 (0.78, 1.25)* | *0.916* |
| *CKD* | *1.12 (0.97, 1.30)* | *0.130* | *1.05 (0.90, 1.23)* | *0.531* |
| *Charlson index* |  |  |  |  |
| *Mild (1-2)* | *Reference* | *Reference* | *Reference* | *Reference* |
| *Moderate (3-4)* | *0.96 (0.82, 1.11)* | *0.563* | *0.99 (0.83, 1.17)* | *0.909* |
| *Severe (5+)* | *0.70 (0.43, 1.10)* | *0.145* | *0.75 (0.44, 1.22)* | *0.266* |
| *BMI (≥ 30)* | *1.29 (1.11, 1.50)* | *<0.001* | *1.10 (0.93, 1.29)* | *0.266* |
| *Smoke (Yes)* | *1.10 (0.86, 1.39)* | *0.431* | *0.85 (0.65, 1.08)* | *0.190* |
| *Hba1c (≥ 7)* | *1.74 (1.51, 2.02)* | *<0.001* | *1.80 (1.55, 2.10)* | *<0.001* |
| *N. Concomitant therapies* |  |  |  |  |
| *1-4* | *Reference* | *Reference* | *Reference* | *Reference* |
| *5-9* | *0.94 (0.80, 1.11)* | *0.482* | *0.92 (0.75, 1.12)* | *0.405* |
| *10+* | *1.08 (0.92, 1.26)* | *0.3* | *1.14 (0.90, 1.45)* | *0.270* |
| *Therapeutic class* |  |  |  |  |
| *Antiplatelets* | *0.99 (0.87, 1.14)* | *0,935* | *0.93 (0.78, 1.10)* | *0.399* |
| *ACE /ARNI inhibitors* | *0.98 (0.85, 1.13)* | *0,800* | *0.90 (0.75, 1.08)* | *0.247* |
| *ARBs* | *1.06 (0.92, 1.22)* | *0,428* | *1.06 (0.89, 1.26)* | *0.530* |
| *Beta blockers* | *1.13 (0.99, 1.30)* | *0,070* | *1.07 (0.91, 1.26)* | *0.412* |
| *Diuretics* | *0.86 (0.73, 1.02)* | *0,082* | *0.83 (0.68, 1.01)* | *0.070* |
| *Lipid lowering agents* | *1.26 (1.10, 1.44)* | *<0.001* | *1.26 (1.07, 1.49)* | *0.005* |

*CKD: patients with a diagnosis code of Chronic kidney disease or patients with eGFR ≤90 ml/min.*

**FIGURE 1.** Percentages of subjects eligible to EMPA-REG OUTCOME, CANVAS, DECLARE-TIMI 58, or VERTIS CV among those treated with respective SGLT2is.


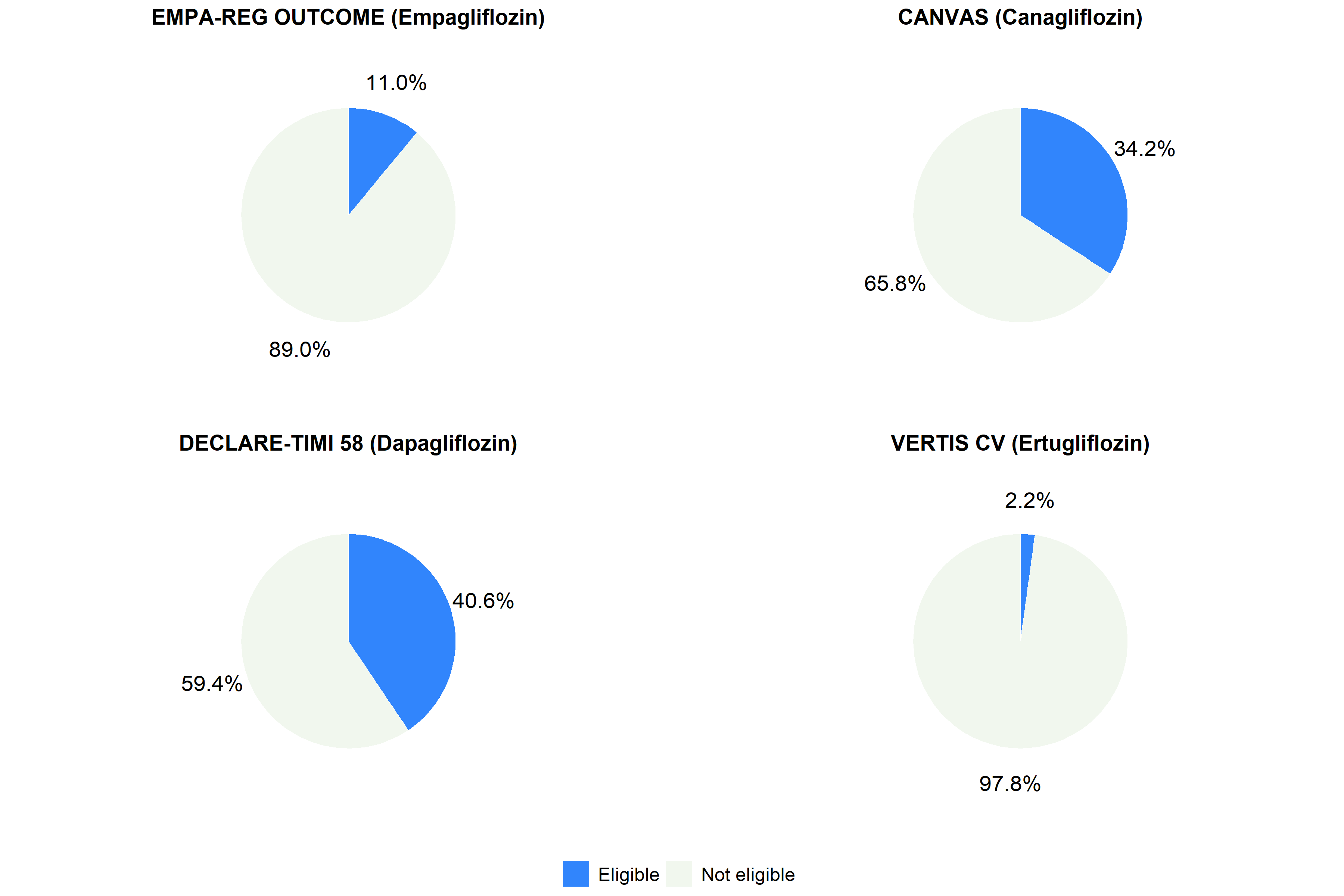

Supplement: Supplementary file 1 — Supplementary Material 1 [file 592_2024_2359_MOESM1_ESM.docx]
